# Advances in Predictions of Oral Bioavailability of Candidate Drugs in Man with New Machine Learning Methodology

**DOI:** 10.3390/molecules26092572

**Published:** 2021-04-28

**Authors:** Urban Fagerholm, Sven Hellberg, Ola Spjuth

**Affiliations:** 1 Prosilico AB, Lännavägen 7, SE-141 45 Huddinge, Sweden; sven.hellberg@prosilico.com (S.H.); ola.spjuth@farmbio.uu.se (O.S.); 2 Department of Pharmaceutical Biosciences and Science for Life Laboratory, Uppsala University, Box 591, SE-751 24 Uppsala, Sweden

**Keywords:** absorption, ADME, bioavailability, computational, in silico, PBPK, pharmacokinetics, prediction, QSAR

## Abstract

Oral bioavailability (F) is an essential determinant for the systemic exposure and dosing regimens of drug candidates. F is determined by numerous processes, and computational predictions of human estimates have so far shown limited results. We describe a new methodology where F in humans is predicted directly from chemical structure using an integrated strategy combining 9 machine learning models, 3 sets of structural alerts, and 2 physiologically-based pharmacokinetic models. We evaluate the model on a benchmark dataset consisting of 184 compounds, obtaining a predictive accuracy (*Q^2^*) of 0.50, which is successful according to a pharmaceutical industry proposal. Twenty-seven compounds were found (beforehand) to be outside the main applicability domain for the model. We compare our results with interspecies correlations (rat, mouse and dog vs. human) using the same dataset, where animal vs. human-correlations (*R^2^*) were found to be 0.21 to 0.40 and maximum prediction errors were smaller than maximum interspecies differences. We conclude that our method has sufficient predictive accuracy to be practically useful with applications in human exposure and dose predictions, compound optimization and decision making, with potential to rationalize drug discovery and development and decrease failures and overexposures in early clinical trials with candidate drugs.

## 1. Introduction

Oral bioavailability (F) is one of the most essential parameters determining the systemic exposure profile and dosing regimens of orally administered drug candidates. Accurate prediction of this parameter is crucial for accurate and safe dosing in first human trials with new drug candidates and for successful drug development and pharmacokinetic (PK) optimization. Overprediction of F might result in insufficient and varying systemic exposure and failure in the clinical phase, and significant underprediction might lead to unwanted side effects in first dose panels in first-time-in-man studies.

An example of a significant, unexpected underprediction of the systemic exposure of a candidate drug was demonstrated by Fuse et al. [1]. The systemic exposure of the anticancer compound UCN-01 following intravenous administration in an early clinical trial was 5800 times higher than predicted from animal PK data (the allometric scaling approach was applied), which corresponds to ca 1 million-fold expected underprediction of the systemic exposure in case the compound had been given orally.

F is determined by several underlying factors, including gastrointestinal solubility/dissolution and passive and active intestinal permeability, which determine the fraction absorbed (f_abs_), and gut-wall metabolism, intrinsic hepatic metabolic clearance (CL_int_), bile extraction, binding to blood components (such as plasma proteins and red blood cells) and flow characteristics in the intestines and liver. The complex nature and species differences complicate predictions of this parameter.

A predictive accuracy (*Q^2^*; forward-looking predictions) of 0.5 (for logit-transformed data), approximately 40–50%, 60% and 75% of predictions with less than 2-, 3-, and 5-fold errors, respectively, and ca 15% of compounds for which no estimates could be generated, was considered successful for computational (in silico) F-predictions according to a recent study by researchers at AstraZeneca [2]. This outcome was what they achieved for various compounds with an in silico prediction model for F in rats. There was, apparently, a skewness of their prediction method, with overprediction trend at low F and significant underprediction trend at high F.

In silico methods have also been developed for prediction of F in man. Paixão reached 47% and 66% of predictions within 20% and 35% absolute errors and 65% of predictions <2-fold error for a set of 68 compounds (50% of these had observed F (F_obs_) ≥ 65%), respectively [3]. There was, however, a 100-fold maximum prediction error and virtually no predictive power of the model (*Q^2^* = 0.01). Lawless et al. (SimulationsPlus) reached 68% of predictions within 2-fold error, *Q^2^* or retrospective correlation (*R^2^*) (not clear which) of ca 0.15, and significant maximum errors, for a set of 62 selected highly permeable and soluble compounds [4].

The comparably low predictive power found for human F-prediction models highlights the need to develop new computational methods with fair/better accuracy and wider compound range. Improved methodology could improve not only early drug candidate selection and optimization, but potentially also enable more accurate and safer dosing in early clinical trials, reduce failures in drug development, reduce the use of animal studies and data, and decrease costs and time-consumption.

Our group has developed an in silico machine learning-based prediction system with fully validated models for predictions of a range of primary and secondary parameters of absorption, distribution, metabolism, excretion/pharmacokinetics (ADME/PK) in humans. In this manuscript we applied this novel methodology to predict in vivo oral F (F_pred_) in humans and compare the results to corresponding F_obs_.

The main aim of the study was to determine whether it is possible to develop a computational system for F in humans with fair true predictive power (according to industry quality standard, as proposed by AstraZeneca; see above) and better outcome than previously developed and published methods. A secondary objective was to compare the results with interspecies correlations—F_obs_ in commonly used laboratory animal models (mice, rats and dogs) vs. F_obs_ in humans. Animal models still appear to be the golden standard for laboratory method-based human ADME/PK-predictions.

The results show that it was possible to develop a new, integrated computational system with sufficient predictive accuracy to be practically useful with applications in human exposure and dose predictions, compound optimization and decision making and that moves the research front forward. The advances also demonstrate that computational systems can outperform and replace most commonly used animal models and potentially also reduce risks and failures in early clinical trials with candidate drugs.

## 2. Results

**Figure 1:**
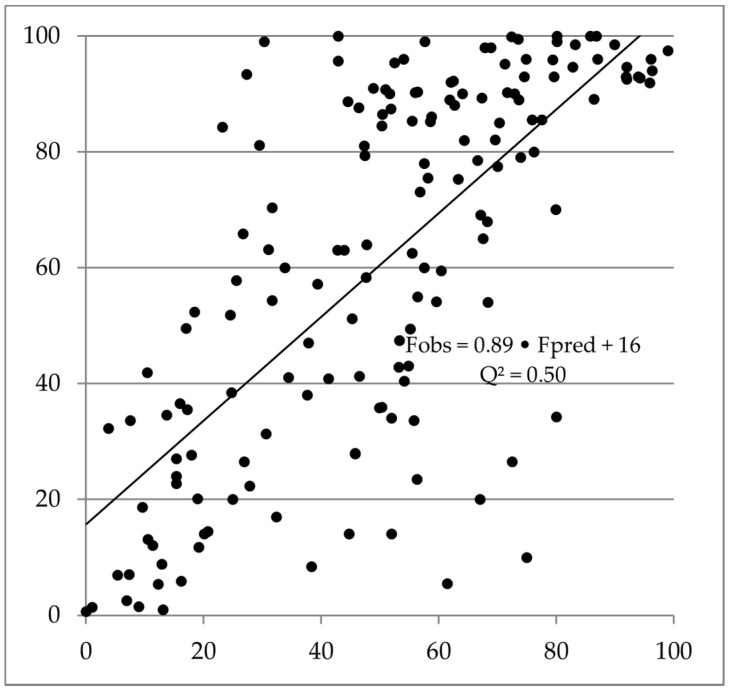
In silico predicted vs. observed human clinical oral bioavailability for 156 compounds.

The *Q^2^* of the new methodology for a diverse dataset was 0.50 (lin-lin scale; *n* = 156; Table 1; Figure 1), which can be considered fairly good and successful.

**Figure 2:**
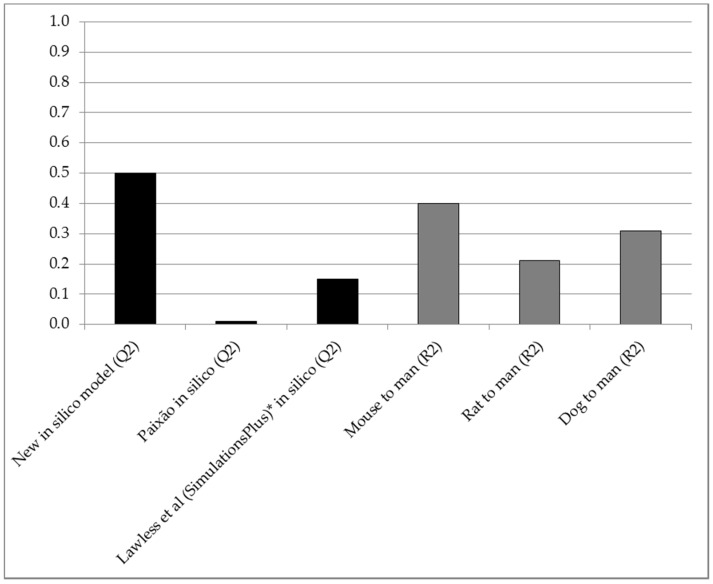
*Q^2^* (predictive accuracy; Y-axis) obtained with the new in silico model (*n* = 156), compared to interspecies R^2^ for compounds of the same dataset (*n* = 28, 101 and 106 for mice, rats and dogs, respectively) and *Q^2^* achieved by other in silico method developers (*n* = 68 and 62 in studies by Paixão and Lawless et al., respectively) [3,4]. * Using a set of compounds with known high permeability, solubility and gastrointestinal absorption [4].

The *R^2^*-estimates between animal and human F_obs_ were lower: 0.40 for mice (*n* = 28), 0.21 for rats (*n* = 101) and 0.31 for dogs (*n* = 106) (Table 1; Figure 2; Supplement Figure S1). Corresponding *Q^2^*-estimates for head-to-head (same compounds) in silico predictions of F in humans were 0.54, 0.61 and 0.48, respectively. Combining data from the three animal species did not enhance the *R^2^* (0.23; *n* = 26).

Figure 2 shows the *Q^2^* for the new in silico model compared to interspecies *R^2^* and *Q^2^* for in silico methods developed and tested by others [3,4].

The benchmark dataset with F_obs_-data for 184 different compounds in humans and animals (see Materials and Methods) was taken from Musther et al. [5].

With this set of data, where the majority of compounds had high human F_obs_ (28 and 50% of human F_obs_-estimates were above 90 and 65%, respectively), there was an intercept for F_pred_ vs. F_obs_ of 16%. For animal models, there was an underestimation trend at low human F_obs_ (ca 5%, 15% and 20% for mice, rats and dogs, respectively) and overestimation trend at high human F_obs_ (ca 30%, 40% and 20% for mice, rats and dogs, respectively).

Forty-eight % of in silico predictions had an absolute error of maximally 15%. For compounds in the sets for which mouse, rat and dog data are available numbers were 57%, 49% and 47%, respectively. Corresponding estimates for interspecies differences were 36% (mouse), 29% (rat) and 51% (dog), respectively.

Three compounds with F_obs_ < 15% were predicted to have an F of >50%. No compound with F_pred_ < 15% had an F_obs_ > 50%; 86% of compounds with an F_pred_ > 40% had an F_obs_ > 40%, and 69% of compounds with an F_pred_ < 15% also had an F_obs_ < 15%.

The median absolute prediction error was 16% (= 1.4-fold relative median error), and the maximum fold-error was 30-fold (for the low permeable risedronate; observed f_abs_ = 1% and predicted f_abs_ = 0.02%). The maximum absolute interspecies differences were greater than the maximum absolute prediction error of the in silico method, 85–92%. The maximum x-fold F-difference for animals vs. man was 6-fold greater than the maximum prediction error of the in silico model.

Eight compounds had a prediction error of >50% (52–69%)—glaziovine (base; phenol; predicted CYP3A4-substrate and MDR-1-substrate; 3.6-fold underprediction), guanfacine (base; MDR-1-substrate; 2.8-fold underprediction), isosorbide-2-mononitrate (neutral; predicted CYP3A4-substrate; 2.3-fold underprediction) isosorbide-5-mononitrate (neutral; predicted CYP3A4-substrate; 2.2-fold underprediction), selegiline (base; CYP3A4-substrate; 7.5-fold overprediction), tetrabenazine (base; predicted CYP3A4-substrate and MDR-1-substrate; 11-fold overprediction), tinidazole (base; CYP3A4-substrate; uncertainty regarding prediction of substrate specificity for MDR-1 and BCRP; 3.3-fold underprediction) and zalcitabine (base; CYP3A4-substrate; 3.4-fold underprediction). A significant portion of selected compounds were substrates for CYP3A4 and/or MDR-1, and the F for many of these was well predicted.

## 3. Discussion

Results clearly show that it is possible to develop an in silico-system with fair/successful predictive accuracy for F in man, and with broad applicability (achieved despite a significant number of compounds with complex ADME/PK) and better performance than earlier models that have been developed for the purpose (both lab- and in silico-based).

According to a standard set at AstraZeneca, the *Q^2^* and % of predictions with <2-, 3-, and 5-fold errors of the new in silico system (0.50, and 77%, 90% and 96%, respectively) is considered acceptable/successful (acceptance/success-limits at AstraZeneca set to 0.50 (logit-transformed) and 40–50%, 60% and 75%, respectively) [2]. The animal prediction model that was considered to be adequate by AstraZeneca had a higher degree of skewness than demonstrated for the new human model. In our own internal validation studies with several hundred compounds we reached a *Q^2^* of 0.54 (reproduced in the current study) and an intercept of only a few %.

Within the main applicability domain for the new in silico methodology (including a molecular weight range of 100 to 700 Da), and with the pre-selected, varied dataset, it outperformed three common animal models (mouse, rat and dog) for predicting human clinical F (assuming that the true predictive performances of animal models match the retrospective fits). The *Q^2^* was clearly higher than corresponding interspecies correlations (Figure 1 and Figure 2; Table 1; Supplement Figure S1) and median and maximum absolute errors were smaller and systematic error was less pronounced.

If an F_obs_ of 20% in two or three animal species had been used to determine whether or not a compound is likely to reach minimum acceptable F_obs_ of 20% in humans, and to select and opt out candidate drugs for clinical development, an incorrect stop-decision (insufficient F in animals; sufficiently high F in humans) would have occurred in 42% of cases. This is 2.5-fold as many as would have been found if the new in silico method had been applied. This, and the 6-fold lower maximum underprediction error, imply improved stop/go-decisions for and reduced overexposure risks in first-time-in-man studies with the in silico method (compared to animal models).

The performance was also better than for in silico methods developed by others [3,4]. Paixão reached a *Q^2^* of 0.01, 47% and 66% of predictions within 20% and 35% absolute errors, respectively, 65% of predictions <2-fold error and a 100-fold maximum prediction error with a dataset of 68 compounds [3]. Lawless et al. (SimulationsPlus) reached a *Q^2^* or *R^2^* (not clear which) of approximately 0.15, 68% of predictions within 2-fold error and maximum prediction errors >30% with a set consisting of 62 selected highly permeable and soluble, non-disclosed compounds [4]. (Note: apparently, as demonstrated by both clinical data and predictions, the majority of modern small drugs do not have high gastrointestinal permeability and/or solubility. Therefore, it is likely that the predictive accuracy of the model by Lawless et al. would be significantly lower than 0.15 if incompletely absorbed compounds had been added to a validation set). With the new in silico methodology and a larger and more diverse dataset (*n* = 156) we reached 60% and 85% of predictions within 20 and 35% absolute errors, respectively, 77% of predictions < 2-fold error and a 30-fold maximum prediction error.

The superior performance of the new system was achieved despite the significant portion of compounds with properties that are challenging/problematic for in vitro labs and in silico methods, such as significant gut wall metabolism, bile excretion, intestinal efflux and active intestinal uptake, high metabolic stability, and low solubility/dissolution potential.

Approximately 20% of the compounds in the dataset has significant first-pass metabolism in the gut wall (many phenols and CYP3A4-substrates, including midazolam, saquinavir and sildenafil). A 36-fold prediction error of the fraction escaping gut wall extraction shown for saquinavir in an in vitro study demonstrated the challenge for in vitro methodology to quantify and predict it [6]. With the new in silico model a 3.7-fold prediction error was obtained for this compound.

Excretion via bile and enterohepatic circulation are also challenging to predict. The dataset contains ca 30% of compounds excreted via bile and reabsorbed by the intestines (including fluvastatin and rosuvastatin).

Predictions of F for compounds with both limited passive permeability and active transport (for example efflux by MDR-1, BCRP and MRP-2 and influx by PEPT-1) and for substances with very high lipophilicity and low solubility/dissolution potential are also challenging for labs.

Nearly every other compound in the dataset has, or is predicted to have, significant active intestinal influx/efflux (including many antibiotics with significant active uptake). Results show that the new in silico methodology has adequate predictive power also for this group of substances.

The 30-fold underprediction of the f_abs_ and F for the low permeability compound risedronate can be compared to maximum prediction errors found for Caco-2-permeability-based predictions. For example, in a Caco-2-study by Matsson et al. there was an approximately 60-fold overprediction of the f_abs_ for raffinose [7]. A *R^2^* of ca 0.6 (predicted vs. observed f_abs_) was obtained for a set of 30 passively absorbed compounds without significant in vivo solubility limitations in that study. In another Caco-2 study with 63 soluble compounds with and without significant efflux the *R^2^* was approximately 0.3 [8]. This is lower than *Q^2^*-values for both F and f_abs_ in the present study.

The selected dataset contains a portion of substances from the top list of low solubility compounds (including azathioprine, felodipine, itraconazole and nifedipine), and their f_abs_ was also overall well-predicted using the new in silico system. Non-specific binding to plastic devices and cells, for example in the Caco-2 assay, is an obstacle for permeability-screening of many candidate drugs. It has been demonstrated by Skolnik et al. that 1/8 and 1/2 of compounds have Caco-2-recoveries below 30% and 80%, respectively, and that this is most pronounced for sticky, lipophilic compounds [9]. According to the authors, poor recovery is often neglected in data interpretation. Apparently, results for problematic compounds are often excluded from analyses of the performance of permeability-screening methods. The new in silico system is devoid of this limitation and predicts for permeability- and solubility/dissolution-based uptake of highly hydrophilic to highly lipophilic substances. It predicts the in vivo dissolution potential with high accuracy (*Q^2^* = 0.57). The *Q^2^*-estimates of models for f_abs,p_, f_diss_, log CL_int_, log f_u_, CL_bile_ and C_bl_/C_pl_ were 0.8, 0.6, 0.5, 0.7, 0.45, 0.8, respectively, and the correctness of transporter (MDR-1 and BCRP) and enzyme (CYP3A4) specificities was 0.85, 0.85 and 0.6, respectively.

Approximately 40% of the compounds in the dataset is estimated to have too high metabolic stability for successful quantification and prediction with conventional in vitro metabolism assays. For example, the CL_int_ of ondansetron, prednisolone, theophylline and metformin could not be quantified with human hepatocytes, and flumazenil, lidocaine, metoclopramide, naloxone and prazosin had non-quantifiable CL_int_ with human microsomes [10,11]. The limit of quantification of the conventional hepatocyte assay corresponds to an in vivo CL_int_ of approximately 500–8000 mL/min (approximately 50% and 80% of marketed drugs have an in vivo CL_int_ below 500 and 5000 mL/min, respectively) [11]. With the new in silico methodology it is possible to generate CL_int_-estimates below 5 mL/min, which is a major advantage over laboratories. Examples of metabolically stable compounds include atenolol (in vivo CL_int_ = 5 mL/min), cimetidine (in vivo CL_int_ = 6 mL/min) and metformin (in vivo CL_int_ = 20 mL/min). With the hepatocyte assay the in vivo CL_int_ of these compounds were overpredicted by approximately 80-, >1000-, and ≥200-fold, respectively [10]. The selected dataset includes metformin. Its in vivo was overpredicted by 10-fold using the new in silico methodology.

Thus, in vitro labs are expected to meet challenges with quantifying and predicting ADME/PK of a significant portion of compounds in the selected dataset. The new in silico system can be utilized to generate missing values in such cases.

The new in silico methodology also has limitations. Absolute prediction errors of 52–69% for F found for some compounds need to be highlighted. These compounds are mainly confirmed and/or predicted CYP3A4-substrates and anticipated to undergo first-pass extraction in the gut-wall. Some of them are also confirmed and/or predicted MDR-1-substrates. The maximum absolute interspecies differences were, however, greater than the maximum absolute prediction error of the in silico model, 85–92%.

There are groups of compounds for which the in silico model does not work (metals and quaternary amines), have limited use (hydrolysis sensitive compounds, for which metabolism cannot be well predicted) and is more uncertain (compounds with molecular weight <100 and >700 Da) and that have the potential/capacity to modulate blood-flows and PK (changed metabolism). In such cases, additional laboratory data are required or useful in order to generate PK data for clinical predictions.

Out of 184 compounds in the dataset, 27 compounds (15%) were outside the main applicability domain for the F-model. Estimates could, however, be generated for all except 2 compounds (a metal and a quaternary amine). For 20 of the 27 compounds excluded from the F-analyses f_abs_ could be predicted well (*Q^2^* = 0.77) (Supplementary Table S2). The percentage of compounds outside the main applicability domain was equal to the percentage of non-predictable found for the rat model developed at AstraZeneca (15%) [2], but lower than for the conventional hepatocyte model (48% in a study by Stringer et al. [11]) and various laboratory methods used within the pharmaceutical industry (16–82%) [12]. In validation and correlation studies of in vitro methods, the portion of challenging compounds is typically kept low. In hepatocyte prediction studies by Sternbeck-Sohlenius et al. and Yamagata et al. only approximately 5–10% of selected reference compounds had low in vivo CL_int_ (compared to approximately 50–80% of marketed drugs) and in vitro CL_int_ (< limit of quantification) [10,13]. In Caco-2-studies by Matsson et al. and Lin et al., compounds with low solubility/dissolution potential were excluded, and the former of these studies was done without including compounds with significant active transport [7,8].

The new in silico model is very useful in cases where laboratories fail, and for predicting when laboratory methods are likely to have problems and fail. This, and the overall higher predictive accuracy vs. animal data based predictions, is believed to have significant impact on predictions of exposures and doses in early clinical studies with new drug candidates.

## 4. Materials and Methods

Here we describe a new model where F_pred_ in humans is predicted directly from chemical structure using support vector machine (SVM) and partial-least squares (PLS) models, new algorithms and a new integrated physiologically-based pharmacokinetic (PBPK) system. We applied it on a benchmark dataset consisting of 184 compounds and compared its performance vs. published dataset of in vivo F_obs_ in mice, rats and dogs vs. humans [5].

### 4.1. Feature Extraction

The core of our method for prediction of F_pred_ in humans consists of a set of 14 models integrated to one (see Table 2).

### 4.2. Quantitative Structure–Activity Relationship (QSAR)/Support Vector Machine (SVM) Model Development

For Models M1-M3, the quantitative structure–activity relationship/support vector machine (QSAR/SVM) models were developed using the Bioclipse software package [14,15] with chemical structures represented using signature descriptors [16], machine learning modeling using a SVM via the libSVM package [17].

A *radial basis function* (RBF) kernel was used and a g grid search was performed to estimate the gamma and cost parameters for SVM optimizing for accuracy in a 10-fold cross validation (CV) [18]. A 10-fold cross validation was performed to assess model performance.

For Models M4-M9, QSAR/PLS modelling was applied to develop regression-like models for the different ADME-related endpoints using signature descriptors, and partial least square discriminant analysis (PLS-DA) for classification models (SIMCA 16 [19]). The predictive power of the resulting models was validated by a 10-fold cross validation procedure, which we normally use in our work and which also appears to be common/default in this field.

M1–M9 (with 4 to 8 components/dimensions) were trained either with QSAR/PLS or QSAR/SVM, not both, and hence there is only one result per model. The application domains for these models were beyond that of the proposed benchmark set for validation of new experimental techniques or in silico models—log P −6.4 to 7.6, log D −10.6 to 12.3, 0 to 19 hydrogen bond donors, and 0 to 19 hydrogen bond acceptors [20].

For Models M10-M12, the structural alerts, based on ocular analyses of the molecular fragments (phenol groups) and known compound class (quinolones, beta-lactam antibiotics), were implemented.

Models M13–14 are PBPK models used for integration of Models M1-M12. The gastrointestinal absorption and extraction model (M13) takes the maximum solubility/dissolution potential in the human gastrointestinal tract following oral dosing (f_diss_; M5), passive permeability-based fractional absorption (f_abs,p_; M1), active uptake (for quinolones and beta-lactam antibiotics; M11 and M12), efflux (by MDR1 and BCRP; M6 and M7), CL_int_ (M2) of CYP3A4-substrates (M3) and phenols (M10) in vivo into consideration for prediction of the fraction of dose absorbed from the intestines and transported unchanged across the gut-wall (F_int_).

The subsequent liver extraction model (M14), based on the classical well-stirred model with a liver blood flow rate of 1500 mL/min, CL_int_ (M2), unbound fraction in plasma (f_u_; M4), blood-to-plasma concentration ratio (C_bl_/C_pl_; M9; used together with f_u_ to estimate the unbound fraction in blood) and biliary CL (CL_bile_; M8), is used to predict the fraction of compound entering the liver that escapes first-pass extraction by the liver (F_liver_). Finally (the integrated model for F_pred_), F_pred_ = F_int_ × F_liver_, where F_int_ = f_abs_ (considering f_diss_ and passive uptake and active transport) × fraction escaping gut-wall extraction (M13) and F_liver_ = (hepatic CL + CL_bile_)/liver blood flow (M14).

All predictions were forward-looking predictions, where compounds were treated as unknown for the models used.

### 4.3. Data Collection

The dataset collected and analyzed for the study consisted of F_obs_ in humans and animals for 184 compounds with varying physicochemical and PK properties (from Musther et al. [5]; see Supplementary Table S1)—bases, neutrals, acids, zwitterions, antibiotics (many with significant active intestinal uptake), phenols (often significant gut-wall extraction), substrates for efflux transporters, CYP3A4 and bile excretion, log P ranging from -6 to 5, f_abs_ ranging from 1–2% (the low permeability compounds risedronate and acarbose) to 100% (e.g., diazepam), f_u_ ranging from 0.1% (naproxen) to 100% (e.g., ifosfamide), CL_int_ ranging from 5 mL/min (fluconazole) to ca 100,000 mL/min (saquinavir), and low in vivo f_diss_ (azathioprine, felodipine, itraconazole and nifedipine) to high solubility/dissolution potential (e.g., zolpidem). The majority of compounds had high human f_abs_ (78% of compounds had an f_abs_ of 80% or greater) and F_obs_ (28 and 50% of human F_obs_-estimates were above 90 and 65%, respectively).

For quality control, F_obs_-data in the dataset were compared to those obtained by others [21]. Apparently, erroneous F_obs_-values (f_abs_ is sometimes mistaken for F) were replaced, and in case sources contained significantly different estimates a mean value was calculated and used.

Twenty-seven of the 184 compounds were excluded from the analysis beforehand as these are outside the main applicability domain for the F-model. These are groups of compounds for which it does not work (metals and quaternary amines), have limited use (cannot predict the stability, f_abs_ and F of hydrolysis sensitive compounds) and is more uncertain (compounds with molecular weight <100 and >700 Da) and that have the potential/capacity to modulate blood-flows and (indirectly) also the PK (such as calcium-blocking agents) (Supplement Table S2). M1–M9 were developed based on available human data for marketed drugs not excluded due to these properties. One compound was excluded because of non-applicable available SMILES (simplified molecular-input line-entry system) (Supplementary Table S2).

For 28, 101 and 106 of the selected compounds with human F_obs_-estimates, F_obs_ data were also available in mice, rats and dogs, respectively.

### 4.4. Performance Evaluation

F_pred_ was compared with F_obs_ on the 156 compounds in the benchmark dataset using *Q^2^* and *R^2^* metrics. Prediction errors, as well as interspecies differences, were presented as median and maximum relative (F_pred_/F_obs_ or F_obs_/F_pred_) and absolute errors (F_pred_-F_obs_ or F_obs_-F_pred_) and % of predictions within certain limits (for example, <15% absolute prediction error). Prediction results were compared with results for interspecies differences, both in total and head-to-head (with same sets of compounds).

## 5. Conclusions

In conclusion, the new in silico methodology had adequate predictive power and range and outperformed animal models within the main applicability domain. It also seems to have significant advantages vs. in vitro methods. Thus, it is now possible to reduce animal use and laboratory studies, improve safety and dose-setting and (potentially also) reduce attrition risk in clinical studies, and obtain sufficiently reliable F-estimates for decision making and optimization already at the drug design stage. This is a major improvement, which also gives significant advantages regarding costs, cost-efficiency, productivity and time.

## Figures and Tables

**Table 1:** Correlations between predicted oral bioavailability (forward-looking in silico predictions) and observed oral bioavailability in animal models (mouse, rat and dog) vs. observed oral bioavailability in man for 156 compounds.

Comparison	All 156 Compounds	28 Compounds with Mouse Data	101 Compounds with Rat Data	106 Compounds with Dog Data
In silico predictive accuracy (*Q^2^*)	0.50	0.54	0.61	0.48
Mouse vs. man correlation (*R^2^*)	-	0.40	-	-
Rat vs. man correlation (*R^2^*)	-	-	0.21	-
Dog vs. man correlation (*R^2^*)	-	-	-	0.31

**Table 2:** The 14 models and algorithms that were integrated to predict oral bioavailability in humans (F_pred_).

Model	Predicted Property	Acronym	Model Type (Number of Components)	Description
M1	Passive intestinal permeability-based fraction absorbed	f_abs,p_	QSAR ^1^/SVM ^2^ (6)	Predicts passive intestinal permeability-based fraction absorbed in vivo in man (not considering active transport, solubility or instability in gastrointestinal fluids)
M2	Intrinsic hepatic metabolic clearance	CL_int_	QSAR/SVM (5)	Predicts intrinsic hepatic metabolic clearance in vivo in man (phase I metabolism and conjugation)
M3	CYP3A4-specificity		QSAR/SVM (5)	Predicts the substrate specificity for CYP3A4 (yes/no)
M4	Fraction unbound in human plasma	f_u_	QSAR/PLS ^3^ (7)	Predicts in vitro fraction unbound in human plasma
M5	Maximum in vivo solubility/dissolution potential	f_diss_	QSAR/PLS (5)	Predicts the maximum solubility/dissolution potential in the human gastrointestinal tract in vivo following oral dosing
M6	MDR-1-specificity		QSAR/PLS-DA ^4^ (4)	Predicts the substrate specificity for MDR-1 (yes/no)
M7	BCRP-specificity		QSAR/PLS-DA (5)	Predicts the substrate specificity for BCRP (yes/no)
M8	Biliary CL	CL_bile_	QSAR/PLS (6)	Predicts the biliary clearance in vivo in man
M9	Blood-to-plasma ratio	C_bl_/C_pl_	QSAR/PLS (8)	Predicts the blood-to-plasma concentration ratio
M10	Phenol detection		Structural alerts	Phenol groups are used for selecting a different method for prediction of gut-wall extraction
M11	Quinolones detection		Structural alerts	Quinolones generally require consideration of active intestinal uptake
M12	Beta-lactam antibiotics detection		Structural alerts	Beta-lactam antibiotics generally require consideration of active intestinal uptake
M13	Intestinal absorption and extraction in the gut-wall		PBPK ^5^	Algorithms for integrating mechanisms involved in intestinal absorption and gut wall extraction (f_abs_, f_diss_, active uptake, efflux by MDR1 and/or BCRP, degradation by CYP3A4 and/or conjugating mucosal enzymes) and prediction of fraction of dose absorbed across the intestinal mucosa
M14	Extraction in the liver		PBPK	Algorithms for integrating mechanisms involved in liver extraction (CL_int_, f_u_, C_bl_/C_pl_, CL_bile_, liver blood flow; well-stirred liver extraction model) and prediction of fraction extracted by the liver

^1^ Quantitative structure–activity relationship (QSAR); ^2^ Support vector machine (SVM); ^3^ Partial least squares regression (PLS); ^4^ Discriminant analysis (DA); ^5^ Physiologically-based pharmacokinetic (PBPK).

## Data Availability

Oral bioavailability data supporting reported results can be found in [5,20] (observed data in humans and animals) and Supplementary Materials (predicted and observed data; Tables S1 and S2 and Figure S1). The integrated method we present partly relies on previously trained QSAR models trained on proprietary data from the Prosilico AB company, and that this training data and used algorithms are not disclosed due to privacy and legal issues.

## Supplementary Materials

The following are available online, Figure S1: Observed oral bioavailability in man vs. mouse, rat and dog, Table S1: Predicted and observed oral bioavailability (F) in man for the selected compounds, Table S2: Compounds excluded beforehand for prediction of oral bioavailability (F) and their predicted and observed fraction absorbed (f_abs_).
