# Supplementary material for: Advances in Predictions of Oral Bioavailability of Candidate Drugs in Man with New Machine Learning Methodology
Source: Molecules. 2021 Apr 28;26(9):2572. doi: 10.3390/molecules26092572 (PMC8124353; doi:10.3390/molecules26092572)
Supplement: Supplementary file 1 [file molecules-26-02572-s001.zip › molecules-1173812-supplementary.pdf]

Supplement
Advances in Predictions of Oral Bioavailability of Candidate Drugs in Man with New Machine Learning Methodology

Urban Fagerholm [1,*], Sven Hellberg [1] and Ola Spjuth [1,2]

[1] Prosilico AB, Lännavägen 7, SE-141 45 Huddinge, Sweden; urban.fagerholm@prosilico.com (U.F.); sven.hellberg@prosilico.com (S.H.); ola.spjuth@farmbio.uu.se (O.S.)
[2] Department of Pharmaceutical Biosciences and Science for Life Laboratory, Uppsala University, Box 591, SE-751 24 Uppsala, Sweden
* Correspondence: urban.fagerholm@prosilico.com; Tel.: +46-70-1731302

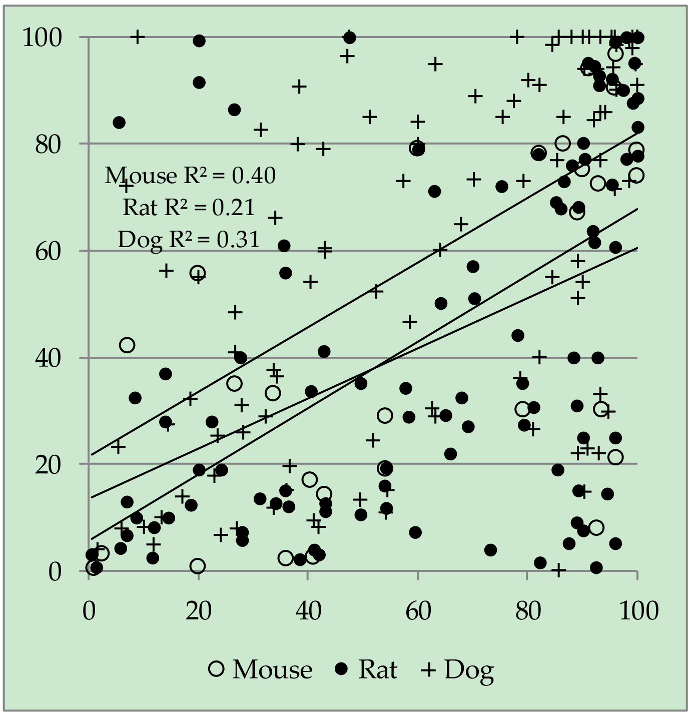

Figure 1. Observed oral bioavailability in humans (x-axis) vs. mouse, rat and dog (y-axis).

Table 1. Predicted and observed oral bioavailability in man for the 156 compounds in the main applicability domain of the model.

**Compound**	**Predicted F (%)**	**Observed F (%) [5,21]**
Acarbose	1.0	1.4
Acebutolol	17.2	35.5
Acyclovir	24.8	38.4
Adefovir	11.4	12.0
Alprazolam	72.9	90.0
Amitriptyline	37.9	47.0
Amoxicillin	58.6	85.2
Antipyrine	89.9	98.5
Azathioprine	56.4	55.0
Cefixime	18.5	52.3
Chlorpheniramine	41.3	40.8
Clonazepam	79.6	93.0
Dapsone	91.9	93.0
Diazepam	94.0	93.0

Doxazosin	47.8	64.0
Estradiol Valerate	16.2	5.9
Ethambutol	51.9	87.4
Ethimizol	38.4	8.4
Ethinylestradiol	10.4	41.9
Ethosuximide	86.8	100.0
Ethylmorphine	45.8	28.0
Etoposide	17.0	49.5
Fenfluramine	67.3	89.3
Fenoterol	9.0	1.5
Fexofenadine	34.5	41.0
Finasteride	76.2	80.0
Fleroxacin	72.4	99.9
Fluconazole	51.7	90.0
Flumazenil	27.9	22.3
Flunisolide	67.0	20.0
Flunitrazepam	70.3	85.0
Fluvastatin	26.9	26.5
Foscarnet	10.5	13.1
Fosfomycin	3.8	32.2
Furosemide	47.7	58.3
Gabapentin	57.5	60.0
Ganciclovir	12.9	8.8
Gatifloxacin	74.9	96.0
Glaziovine	23.2	84.3
Glyburide (Glibenclamide)	71.7	90.2
Griseofulvin	56.8	73.1
Guanfacine	29.5	81.1
Hydralazine	55.8	33.6
Hydrochlorothiazide	31.0	63.1
Ibuprofen	99.0	97.5
Idazoxan	52.0	34.0
Ifosphamide	56.0	90.2
Indapamide	57.7	99.0
Indomethacin	95.9	91.9
Isosorbide dinitrate	18.3	27.6
Isosorbide-2-mononitrate	42.9	100.0
Isosorbide-5-mononitrate	42.9	95.7
Isoxicam	83.2	98.5
Ketanserin	53.3	47.4
Ketorolac	79.4	95.9
Lansoprazole	47.3	81.0
Levodopa	80.0	34.2
Levofloxacin	80.1	99.0
Levonorgestrel	61.9	89.0
Lidocaine	45.8	27.8
Linezolid	73.5	99.5
Lisuride	44.8	14.0
Losartan	49.9	35.8
Melagatran	5.4	6.9
Meloxicam	48.9	91.0
Menogaril	7.5	33.6
Mepindolol	69.6	82.1

Mercaptopurine	51.6	14.0
Metformin	25.6	57.8
Methadone	77.6	85.5
Methylprednisolone	55.2	49.4
Metoclopramide	63.3	75.3
Metolazone	37.6	38.0
Metoprolol	46.5	41.3
Midazolam	50.3	35.9
Morphine	16.3	36.5
Moxifloxacin	64.4	82.0
Moxonidine	46.4	87.6
Nalbuphine	19.2	11.7
Naloxone	13.1	0.9
Naltrexone	24.6	20.0
Naproxen	75.9	85.5
Naratriptan	43.9	63.0
Nefazodone	32.4	17.0
Nevirapine	74.6	93.0
Nizatidine	52.5	95.4
Nomifensine	72.5	26.5
Norfenfluramine	55.5	85.3
Ofloxacin	80.1	100.0
Omeprazole	31.7	54.3
Ondansetron	60.4	59.5
Oseltamivir acid	73.9	79.0
Oxazepam	94.3	92.8
Phenobarbital	96.1	96.0
Phenoxymethylpenicillin (Penicillin V)	59.6	54.1
Phenytoin	66.6	78.5
Pindolol	50.4	86.5
Piroxicam	85.8	100.0
Pravastatin	9.6	18.6
Prazosin	55.5	62.5
Prednisolone	50.3	84.5
Prednisone	44.6	88.7
Primaquine	86.9	96.0
Procainamide	58.2	75.5
Propoxyphene	56.3	23.5
Propranolol (−)	15.4	27.0
Propranolol (+)	15.4	22.7
Propranolol (±)	15.4	24.0
Propylthiouracil	70.4	77.5
Quinidine	79.9	70.0
Rabeprazole	24.6	51.8
Ranitidine	39.4	57.2
Reboxetine	54.0	96.0
Recainam	31.7	70.3
Remoxipride	92.0	92.5
Risedronate	0.02	0.6
Risperidone	26.7	65.9
Rosiglitazone	68.9	98.0
Rosuvastatin	19.0	20.1
Salbutamol	45.3	51.2

Salicylate	71.3	95.2
Saquinavir	7.3	7.0
Selegiline	75.0	10.0
Sildenafil	54.1	40.4
Sitafloxacin	73.6	89.0
Sitagliptin	62.7	88.0
Sotalol	33.8	60.0
Sparfloxacin	62.1	92.0
Sulfisoxazole	67.9	98.0
Sulpiride	30.6	31.3
Sumatriptan	20.1	14.0
Talinolol	67.6	65.0
Tamsulosin	92.0	94.6
Terazosin	58.8	86.0
Terodiline	50.9	90.8
Tetrabenazine	61.5	5.5
Theophylline	56.4	90.3
Tiagabine	64.0	90.0
Timolol	42.8	63.0
Tinidazole	30.3	99.0
Tolterodine	13.7	34.5
Torsemide	82.8	94.6
Tramadol	68.3	67.9
Trazodone	47.4	79.3
Triazolam	68.4	54.0
Trovafloxacin	86.4	89.1
Valproic Acid	62.5	92.2
Vardenafil	20.7	14.5
Warfarin	96.3	94.0
Venlafaxine	54.9	43.0
Xamoterol	12.3	5.3
Zalcitabine	27.3	93.4
Zanamivir	6.9	2.5
Zolmitriptan	53.2	42.8
Zolpidem	67.1	69.1
Zopiclone	57.5	78.0

Table 2. Compounds excluded beforehand for prediction of oral bioavailability (F) and their predicted and observed fraction absorbed (f_{abs}).

Compound	Predicted f_{abs} (%)	Observed f_{abs} (%)	Reason for exclusion beforehand
5-Fluorouracil	79	High permeability	Hydrolysed
Acetylsalicylate	90	82	Prodrug
Amlodipine	100	95	Calcium-blocker; blood-flow modulating
Amosulalol	90	100	Uncertain/questionable human F-estimate
Azithromycin	13	37	MW >700 Da
Cefuroxime axetil	19	34	Prodrug
Cyclosporine	4	40	MW >700 Da
Erythomycin	15	>33	MW >700 Da
Estramustine phosphate	44	82	Prodrug
Felodipine	100	91	Calcium-blocker; blood-flow modulating
Gitoxin	17	?	MW >700 Da
Itraconazole	48	82	MW >700 Da
Lithium carbonate	-	94	Metal (not possible to predict)
Nicardipine	95	95	Calcium-blocker; blood-flow modulating
Nifedipine	100	95	Calcium-blocker; blood-flow modulating
Nimodipine	95	90	Calcium-blocker; blood-flow modulating
Nisoldipine	100	90	Calcium-blocker; blood-flow modulating
Nitrendipine	100	88	Calcium-blocker; blood-flow modulating
Nufenoxole SC-27166	-	100	SMILES did not work (not possible to predict)
Physostigmin	87	?	Hydrolysed
Pyridostigmin	-	?	Quaternary amine (not possible to predict)
Rifabutin	31	53	MW >700 Da
Rifampin	90	95	MW >700 Da
Tacrolimus	33	15	MW >700 Da
TRH tartrate (Protirelin)	12	?	Hydrolysed?
Verapamil (−)	95	100	Calcium-blocker; blood-flow modulating
Verapamil (+)	95	100	Calcium-blocker; blood-flow modulating
Verapamil (±)	95	100	Calcium-blocker; blood-flow modulating
